# The Prognostic Value of Plasma Soluble ST2 in Hospitalized Chinese Patients with Heart Failure

**DOI:** 10.1371/journal.pone.0110976

**Published:** 2014-10-27

**Authors:** Rongcheng Zhang, Yuhui Zhang, Jian Zhang, Tao An, Yan Huang, Xiao Guo, James L. Januzzi, Thomas P. Cappola, Shijie Yin, Yunhong Wang, Qiong Zhou, Changhong Zou, Shiming Ji, Rong Lv

**Affiliations:** 1 State Key Laboratory of Cardiovascular Disease, Heart Failure Center Fuwai Hospital, National Center for Cardiovascular Diseases, Chinese Academy of Medical Sciences and Peking Union Medical College, Beijing, China; 2 Division of Cardiology, Massachusetts General Hospital, Boston, Massachusetts, United States of America; 3 Penn Cardiovascular Institute, University of Pennsylvania School of Medicine, Philadelphia, Pennsylvania, United States of America; Boston University, United States of America

## Abstract

**Background:**

sST2 has been shown to be a risk predictor in heart failure (HF). Our aim was to explore the characteristics and prognostic value of soluble ST2 (sST2) in hospitalized Chinese patients with HF.

**Methods and Results:**

We consecutively enrolled 1528 hospitalized patients with HF. Receiver operating characteristic (ROC) and multivariable Cox proportional hazards analysis were used to assess the prognostic values of sST2. Adverse events were defined as all-cause death and cardiac transplantation. During a median follow-up of 19.1 months, 325 patients experienced adverse events. Compared with patients free of events, sST2 concentrations were significantly higher in patients with events (*P*<0.001). Univariable and multivariable Cox regression analyses showed sST2 concentrations were significantly associated with adverse events (per 1 log unit, adjusted hazard ratio 1.52, 95% confidence interval: 1.30 to 1.78, *P*<0.001). An sST2 concentration in the highest quartiles (>55.6 ng/mL) independently predicted events in comparison to the lowest quartile (≤25.2 ng/mL) when adjusted by multivariable model. In ROC analysis, the area under the curve for sST2 was not different from that for NT-proBNP in short and longer term. Over time, sST2 also improved discrimination and reclassification of risk beyond NT-proBNP.

**Conclusions:**

sST2 is a strong independent risk predictor in Chinese patients hospitalized with HF and can significantly provide additional prognostic value to NT-proBNP in risk prediction.

## Introduction

Heart failure (HF) is the primary diagnosis in hospitalized patients and has become a growing major public health issue [1], [2]. The reported 1-year mortality following hospitalization for acutely decompensated HF is estimated at nearly 30% [3], [4] and the approach to evaluation and management of the HF patient is complex. Accordingly, with increasingly diverse medical and non-medical strategies to treat patients with HF, it is important for physicians to accurately assess the risk of patients in order to tailor their therapies. Determination of circulating biomarkers has been suggested as a meaningful approach to reflect biological process and predict the outcomes in HF. In this regard, over recent years, the natriuretic peptides (including N-terminal pro-B type natriuretic peptide; NT-proBNP) have been well recognized as important risk predictors in HF. However natriuretic peptides alone are insufficient to explain the complexity of pathophysiologic pathways in HF. Therefore, other biomarkers might be useful to improve risk stratification and prognostication for patients with HF.

ST2 is a member of the interleukin (IL)-1 receptor family with transmembrane (ST2L) and soluble (sST2) forms. Clinically, many studies have shown that elevated concentrations of sST2 are associated with adverse events in patients with acute myocardial infarction [5], [6], HF [7]–[11] and dyspnea [12], [13]. Additionally, the ability of sST2 to prognostic was recently found to be superior to another novel biomarker, galectin-3, in patients with chronic HF [14]. Despite these encouraging results, no data exist regarding the prognostic value of sST2 measurement in patients with HF from Asia, a distinctly different ethnic group, with diverse medical issues compared to Western populations. Therefore, the purpose of this study was to investigate the clinical characteristics of sST2 and evaluate the prognostic values in a large cohort of Chinese hospitalized patients with HF.

## Methods

### Study population and design

We consecutively enrolled hospitalized patients identified admitted to FuWai Hospital HF center, Beijing, China from March 2009 to April 2013 with a diagnosis of HF. The diagnosis of HF was confirmed by two specialists according to current guidelines [15], [16]. In this study, hospitalized patients with HF (defined as a *de novo* presentation of HF or worsening of previously chronic stable HF requiring unplanned hospitalization) were evaluated and enrolled if they had HF as their primary diagnosis, had venous blood sample available for biomarkers analysis, and were aged 18 years or older at the time of hospitalization. Patients with a diagnosis of acute coronary syndrome, cancer, acute pulmonary embolism were excluded from this analysis. An ischemic etiology of HF was assumed if the patient had prevalent angina pectoris, or a prior history of coronary artery bypass grafting, percutaneous coronary intervention, acute myocardial infarction, or confirmed coronary artery obstruction by coronary angiography or computed tomography angiography.

Data from medical records are abstracted by trained clinicians or cardiology nurses and were entered into a predefined electronic case report form with checking by another abstractor. Clinical data including demographic characteristics, New York Heart Association (NYHA) functional class, primary HF etiologies, vital signs, and physical examination; also; preexisting comorbidities and medical history were obtained at the time of the hospitalization. All patients received intravenous loop diuretics at least during the first 24 h of admission. Echocardiography was performed to assess left ventricular ejection fraction (LVEF), left ventricular internal diameters at end diastole (LVIDd), posterior and septal wall thickness at end diastole (PWTd and SWTd) within 48 hours after admission, and interpreted by trained echocardiographers. Echocardiographers and other study staff were blinded to sST2 and galectin-3 values. LV mass was calculated with the use of the American Society of Echocardiography-recommended formula: 0.8×(1.04 [(LVIDd + PWTd + SWTd)^3^ – (LVIDd)^3^]) +0.6 g. LV hypertrophy was evaluated by LV mass indexed to body surface area (LV mass index [LVMi]). Relative wall thickness (RWT) was calculated by formula: (2× PWTd)/LVIDd [17].

For patients with multiple admissions, only the first admission was included in this study. Adverse events with respect to all-cause death and cardiac transplantation were ascertained every 3 months via electronic hospital records follow-up or conversations with patients or patients' families by telephone. All patients provided written informed consent and the ethics committee of FuWai Hospital approved the study procedure.

### Biomarker measurement

Fasting venous blood sample were collected within 12 hours of hospitalization, immediately centrifuged and stored at −80°C in plasma. sST2 and NT-proBNP were determined by blood samples subjected to no more than one freeze-thaw cycle. All measurements of these two markers were performed by 3 professional laboratory technicians in the central lab of FuWai hospital. Coefficient of variations for intra-assay and inter-assay were used to qualify the measurements of both biomarkers. Information about these two assays is detailed in Table S1.

## Statistical Analyses

Continuous variables were tested for normal distribution by using Kolmogorov-Smironov test and were described as means ± SD for normally distributed variables and medians and interquartile range (IQR) for variables with skewed distribution. Categorical variables were described as percentages. Comparisons between two groups were performed by Student t-test for symmetrical continuous, Mann-Whitney U test for nonsymmetric continuous, and χ2 tests for categorical variables. Kruskal-Wallis H testing was used to compare more than two groups. Logarithmic transformation was performed to normalize the distribution of NT-proBNP and sST2. Univariable Spearman correlation was used to evaluate the relationships among continuous variables. Multivariable linear regression analyses were then performed with stepwise method, using log-transformed of sST2 levels as the dependent variables respectively.

Following identification of candidate variables associated with adverse events in univariable Cox regression analysis (*P*≤0.10), we proceeded with multivariable analysis. Variables with significant *P* values (*P*<0.05) were retained in the final multivariable model. The prognostic values of sST2 were evaluated by univariable and established multivariable model. sST2 quartiles were also used to estimate the associations of sST2 concentrations with the risk of adverse events. Multicollinearity among covariates were checked and found none of significance. Receiver operating characteristic (ROC) curves were performed to determine the prognostic ability of sST2 for events, and were also used to identify the optimum cut-off points of sST2, using the Youden approach. Log-rank tests for Kaplan-Meier survival curves were used for comparisons. Differences in area under the curve (AUC), net reclassification improvement (NRI) and integrated discrimination improvement (IDI) were performed to evaluate the added predictive value of sST2 when combined with NT-proBNP according to follow up time [18]. As there are no established gold standard risk categories for HF, we used category-free NRI as described by Pencina et al [19]. Confidence intervals and *P* values for NRI and IDI were determined by bootstrapping with 1000 repetitions. All *P* values of less than 0.05 from two-sided tests were accepted as statistically significant. Statistical analyses were conducted using SPSS version 19.0 (SPSS Inc., Chicago, Illinois) and Stata version 11.2 (StataCorp LP, College Station, TX, USA).

## Results

### Baseline characteristics

A total of 1940 patients presented with HF were admitted between March 2009 and April 2013. 412 patients were excluded by design (Figure S1). Of the remaining 1528 patients, 122 patients were lost to follow up, 325 experienced adverse events (300 patients died, 25 patients underwent cardiac transplantation) during a median of 19.1 months follow up. All studied patients were from diffuse geographic regions across China, most of them came from the north (Figure S2).

All available data of the study patients are shown in File S1. Table 1 depicts baseline characteristics of these patients according to outcome. The mean age was 58 years, the majority was male (70.4%), more than half had a history of HF. The proportion of hypertension history (47.3%) in patients with HF was similar to that of ischemic heart disease (47.0%), indicating hypertension might be the main factor associated with ischemic heart disease and HF in China. Furthermore, this population studied consists of a large subset of patients with valvular heart disease (14.1%), which still one of the main causes of HF in Chinese patients. Consistent with current HF statistics, 51.5% of patient had preserved LVEF at admission. The median (IQR) sST2 concentration was 37.1(25.2–56.7)ng/mL.

**Table 1:** Baseline characteristics of study populations according to outcome.

Variables	All patients (n = 1528)	Outcome		
		Patients without adverse events (n = 1203)	Patients with adverse events (n = 325)	*P* value
Age, years	58±16	57±15	59±17	0.003
Male, n (%)	1075 (70.4)	839 (69.7)	236 (72.6)	0.314
History, n (%)				
Hypertension	723 (47.3)	591 (49.3)	132 (40.6)	0.006
Diabetes mellitus	395 (25.9)	303 (25.2)	92 (28.3)	0.254
Ischemic heart disease	718 (47.0)	581 (48.3)	137 (42.2)	0.049
Nonischemic cardiomyopathy	430 (28.1)	316 (26.3)	114 (35.1)	0.002
Valvular heart disease	216 (14.1)	165 (13.7)	51 (15.7)	0.364
Congenital heart disease	48 (3.1)	37 (3.1)	11 (3.4)	0.777
Previous heart failure	1165 (76.2)	891 (58.3)	274 (84.3)	<0.001
Physical examination				
Heart rate, beats/min	78±18	78±17	80±20	0.006
Systolic blood pressure, mmHg	119±20	121±20	111±20	<0.001
Body mass index, kg/m^2^	24.2±4.2	24.6±4.3	22.8±3.5	<0.001
NYHA functional class, n (%)				<0.001
II	457 (29.9)	422 (35.1)	35 (10.8)	
III	635 (41.6)	517 (43.0)	118 (36.3)	
IV	436 (28.5)	264 (21.9)	172 (52.9)	
LVEF (%)	40 (30–56)	42 (30–58)	35 (27–50)	<0.001
LVEF				0.640
LVEF ≤ 40%	787 (51.5)	616 (51.2)	171 (52.6)	
50%> LVEF> 40%	169 (11.1)	130 (10.8)	39 (12.0)	
LVEF ≥ 50%	572 (37.4)	457 (38.0)	115 (35.4)	
LV mass index (g/m^2^)	131.7 (104.2–169.1)	129.2 (102.2–.7)	143.4 (109.0–181.5)	<0.001
Relative wall thickness	0.31 (0.26–0.38)	0.32 (0.26–0.38)	0.30 (0.24–0.37)	0.009
Current smoking, n (%)	376 (24.6)	304 (25.3)	72 (22.2)	<0.001
Medication on presentation, n (%)				
Digoxin	732 (47.9)	547 (45.5)	185 (56.9)	<0.001
Loop diuretics	1013 (66.3)	775 (50.7)	238 (73.2)	0.003
ACEI/ARB	798 (52.2)	657 (54.6)	141 (43.4)	<0.001
Aldosterone antagonists	941 (61.6)	732 (60.8)	209 (64.3)	0.255
β-blockers	1164 (76.2)	931 (77.4)	233 (71.7)	0.032
Laboratory results				
White blood cell count	7.1 (5.8–8.9)	7.1(5.8–8.8)	7.1 (5.9–9.2)	0.212
Hemoglobin, g/dL	133.8±22.6	134.9±21.8	129.6±24.7	0.003
Sodium, mmol/L	139.3±3.5	139.7±3.2	137.9±4.0	<0.001
Total cholesterol, mmol/L	4.15 (3.5–4.97)	4.21 (3.58–5.03)	3.96 (3.23–4.72)	<0.001
High density cholesterol, mmol/L	0.98 (0.82–1.19)	1.0 (0.83–1.21)	0.94 (0.74–1.13)	<0.001
Creatinine, ummol/L	88 (73–109)	86 (72–105)	99 (79–130)	<0.001
Blood urea nitrogen, mg/dL	7.6 (5.8–10.1)	7.2 (5.6–9.5)	9.2 (7–12.7)	<0.001
Uric acid, g/dL	416 (319–522)	402 (312–498)	470 (360–598)	<0.001
Total bilirubin, umol/L	19.5 (13.6–29.3)	18.2(13.3–26.8)	25.7 (18.1–44.4)	<0.001
Albumin, g/L	39.8 (36.6–42.9)	40.2 (37.4–43.1)	37.6 (34–40.9)	<0.001
C-reactive protein, mg/L	4.8 (2.6–11.7)	4.3 (2.3–10.2)	7.5 (3.8–21.0)	<0.001
NT-proBNP, pg/mL	1557 (781–3299)	1308 (703–2668)	3194 (1676–5874)	<0.001
sST2, ng/mL	37.1 (25.2–55.7)	33.6 (24.0–47.3)	55.6 (36.3–103.7)	<0.001

Data are expressed as mean (standard deviation), median [percentiles 25^th^ – 75^th^] or absolute number (percentage).

ACEI  =  angiotension-converting enzyme inhibitor; ARB  =  angiotensin receptor blocker; LVEF  =  left ventricular ejection fraction;

NT-proBNP  =  N-terminal pro-B-type natriuretic peptide; NYHA  =  New York Heart Association.

Patients without blood samples available (n = 197) did not differ in age, gender and LVEF when compared with included patients.

### Distribution and correlations of sST2

**Figure 1:**
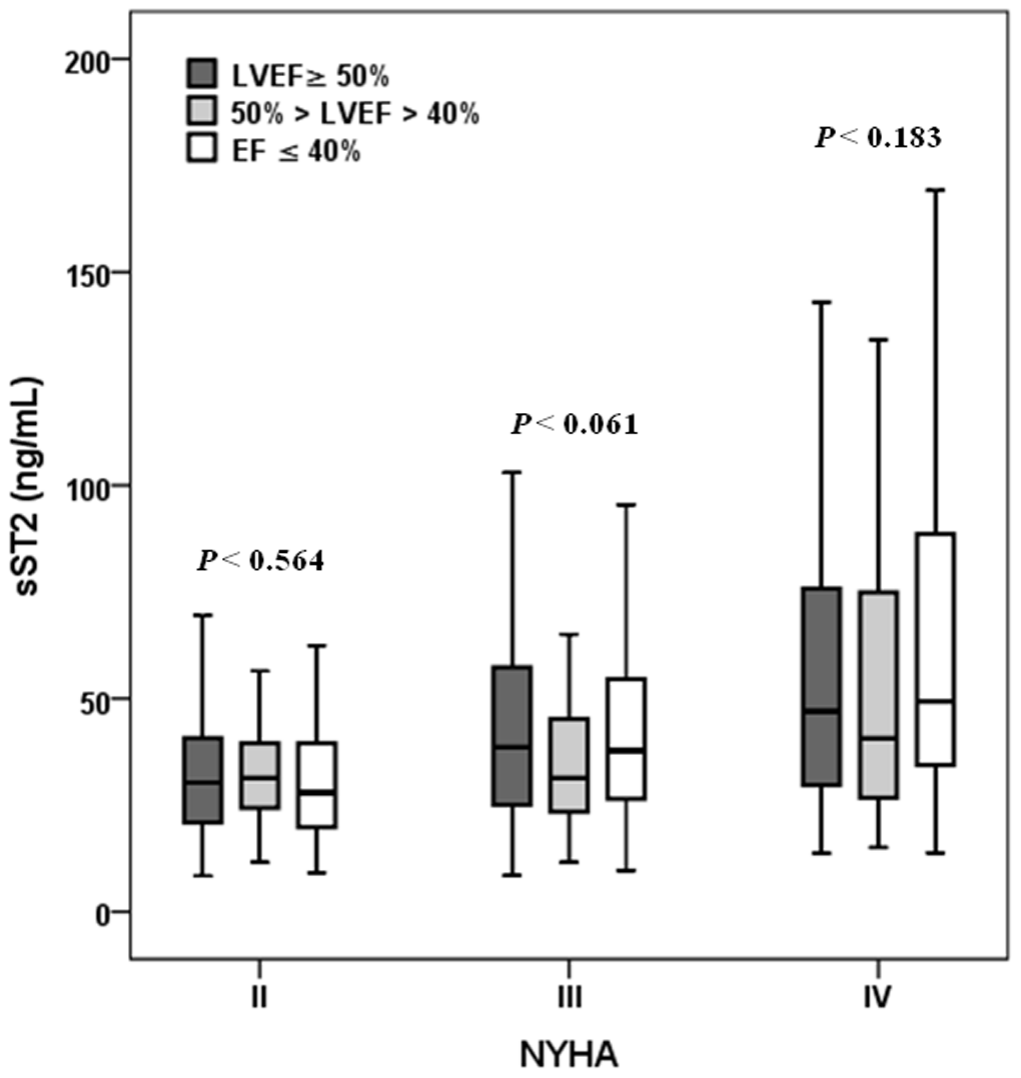
Values of sST2 and associations with left ventricular ejection fraction as a function of New York Heart Association functional class. *P* values indicated the differences among groups stratified by left ventricular ejection fraction.

Median sST2 concentrations were higher in those with worse symptoms defined by NYHA functional class (*P*<0.001). Interestingly, patients with nonischemic HF etiology had a significantly higher sST2 concentration when compared with patients with ischemic heart disease (*P*<0.001), possibly explained by the proportions of patients with NYHA functional class IV being different in those two groups (36.5% vs. 19.9%; *P*<0.001). When LVEF was categorized by using the cut-off points of EF ≥50%, 50%> EF> 40%, EF ≤ 40%, sST2 concentrations were noted to be higher in patients with lower LVEF (*P*<0.001), but no significant difference was detected after regarding NYHA functional class (Figure 1).

Univariable correlations of sST2 with other continuous variables were shown in Table S2. Higher levels of sST2 were correlated with lower LVEF, and severity of renal and liver function. Interestingly, as a biomarker of cardiac fibrosis, sST2 concentrations have a tendency to correlate with LV mass index (*P* = 0.061), but weak and negatively associated with RWT (r = -0.062; *P* = 0.020). A multivariable linear regression model with log-transformed of sST2 as the dependent variables showed sST2 concentrations were predicted by NT-proBNP (T = 9.55; *P*<0.001), total bilirubin (T = 10.44; *P*<0.001), C-reactive protein (T = 5.46; *P*<0.001), white blood cell count (T = 6.24; *P*<0.001), albumin (T = −6.06; *P*<0.001), sodium (T = −4.98; *P*<0.001), blood urea nitrogen (T = 5.85; *P*<0.001), systolic blood pressure (T = −3.80; *P*<0.001), and heart rate (T = 3.72; *P*<0.001). The R^2^ was 0.38.

### Prognosis of all-cause death or cardiac transplantation

**Figure 2:**
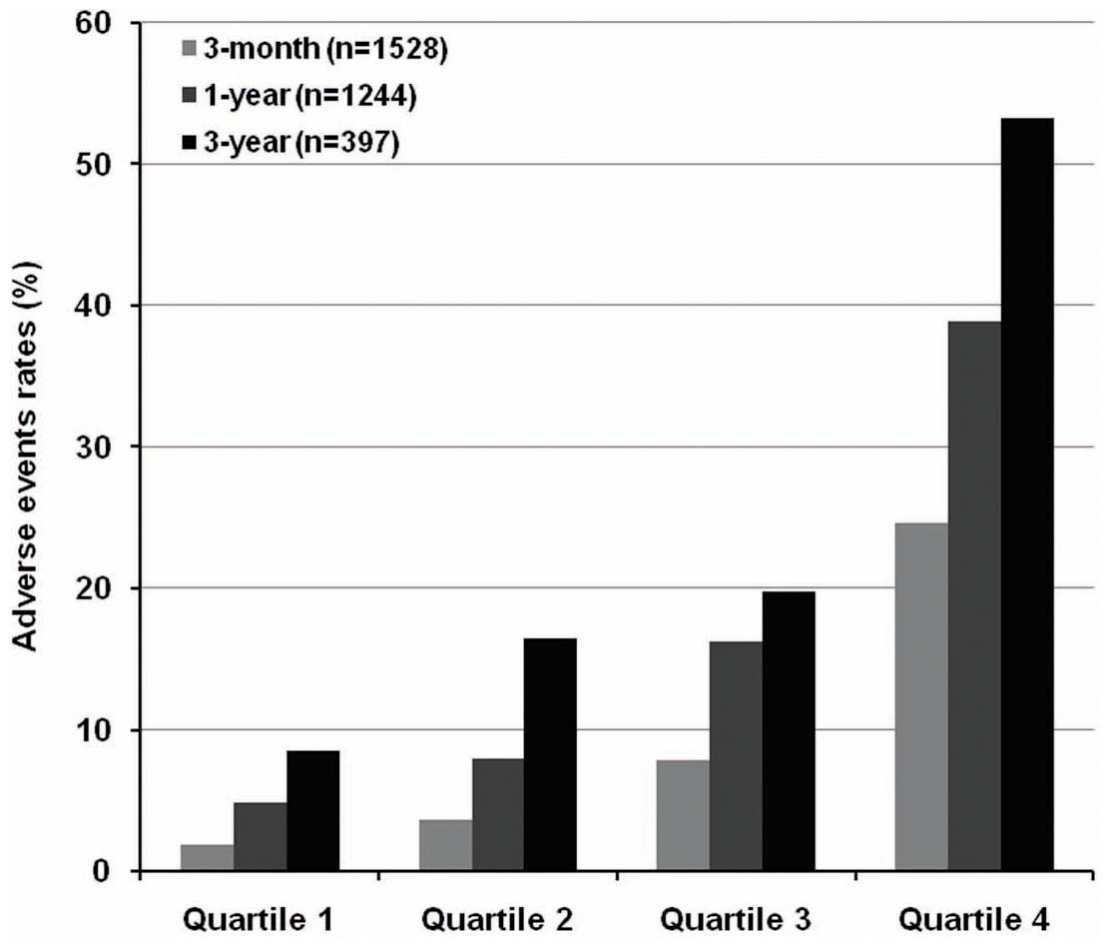
Rate of all-cause death or cardiac transplantation according to sST2 quartiles at 3 months (*P*<0.001 for trend), 1 year (*P*<0.001 for trend) and 3 years (*P*<0.001 for trend).

**Figure 3:**
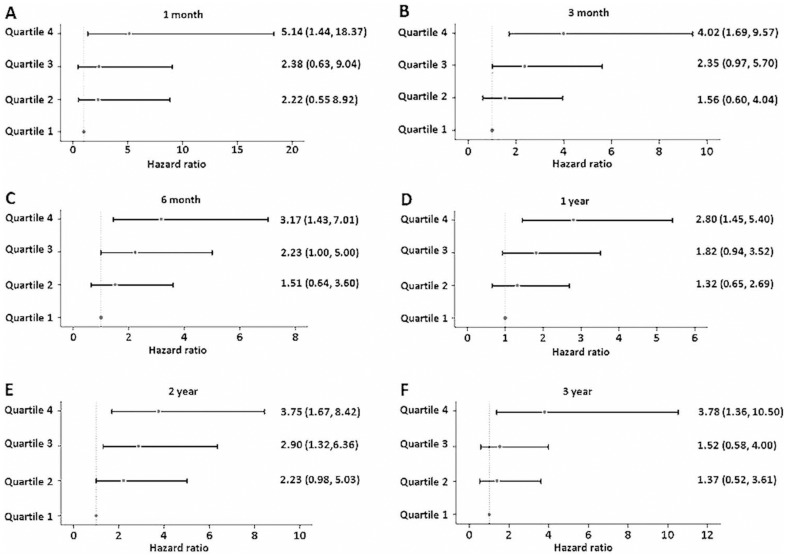
Hazard Ratios for the association between sST2 quartiles and all-cause death or cardiac transplantation according to follow-up time. (A) 1 month; (B) 3 month; (C) 6 month; (D) 1 year; (E) 2 year; and (F) 3 year. Multivariable Cox regression analyses were performed to obtain hazard ratios. Patients in the lowest quartiles were used as reference. Patients with the highest quartiles showed significant hazard ratio for all-cause death or cardiac transplantation in comparison with the patients with the first quartile after adjustment for clinical risk factors (all *P* value <0.001).

**Figure 4:**
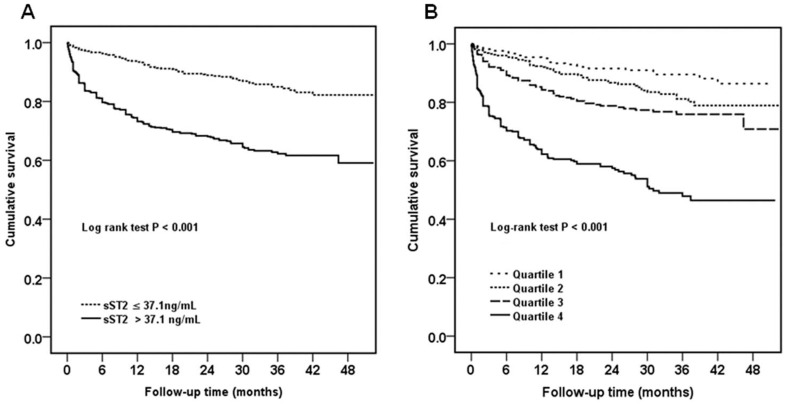
Kaplan-Meier survival curves for all-cause death or cardiac transplantation according to (A) sST2 quartiles, and (B) sST2 median for all patients. *P* values indicated the differences among groups.

Concentrations of sST2 were significantly elevated among patients with adverse events in comparison to patients without adverse events (*P*<0.001). A graded increase was observed in adverse events rates in 3-month, 1-year and 3-year according to sST2 quartiles (Figure 2). Cox regression showed sST2 concentrations were significantly associated with the combined end point in univariable and multivariable analysis after adjustment for age, systolic blood pressure, body mass index, NYHA functional class, left ventricular ejection fraction, sodium, total cholesterol, blood urea nitrogen, total bilirubin, albumin, NT-proBNP and loop diuretics treatment (per 1 log unit, adjusted hazard ratio 1.52, 95% CI: 1.30 to 1.78; *P*<0.001) (Table 2). Patients with sST2 quartiles higher than the first quartile (sST2>25.2 ng/mL) had significant unadjusted hazard ratio (Table 2), and the highest quartiles (sST2>55.6 ng/mL) remained robust in multivariable model compared with the first quartile (adjusted hazard ratio 2.11, 95% CI: 1.39 to 3.21; *P* = 0.001). This significant result remained stable in predicting short term and longer term adverse events according to follow-up time (Figure 3). Kaplan-Meier survival curves depicting time to first event are shown according to sST2 quartile (Fig 4A) and median (Fig 4B).

**Table 2:** Univariable and multivariable Cox regression analysis for predicting all-cause death and transplantation.

Variables	Univariable			Multivariable		
	HR	95% CI	*P* value	HR	95% CI	*P* value
Age, yrs	1.009	1.001–1.016	0.022*	1.01	1.004–1.019	0.002†
Male	1.13	0.89–1.45	0.32	-	-	-
Current smoking	0.80	0.61–1.04	0.091*	-	-	-
Hypertension	0.72	0.57–0.89	0.003*	-	-	-
Diabetes mellitus	1.15	0.91–1.47	0.25	-	-	-
Ischemic heart disease	0.76	0.61–0.95	0.016*	-	-	-
Previous heart failure	1.90	1.41–2.56	<0.001*	-	-	-
Heart rate, beats/min	1.009	1.003–1.02	0.004*	-	-	-
Systolic blood pressure, mmHg	0.971	0.965–0.977	<0.001*	0.986	0.979-0.993	<0.001†
Body mass index, kg/m^2^	0.90	0.87–0.92	<0.001*	0.96	0.93–0.99	0.005†
NYHA functional class						
III (vs. II)	2.70	1.85–3.93	<0.001*	1.63	1.10–2.43	0.015†
IV (vs. II)	6.63	4.61–9.54	<0.001*	2.72	1.82–4.08	<0.001†
Left ventricular ejection fraction (%)	0.97	0.97–0.98	<0.001*	0.99	0.98–1.00	0.021†
Digoxin	1.49	1.19–1.85	<0.001*	-	-	-
Loop diuretics	1.52	1.19–1.94	0.001*	0.72	0.55–0.95	0.019†
ACEI/ARB	0.60	0.48–0.75	<0.001*	-	-	-
Aldosterone antagonists	1.17	0.93–1.47	0.180	-	-	-
β-blockers	0.69	0.54–0.88	0.002*	-	-	-
White blood cell count	1.05	1.02–1.09	0.002*	-	-	-
Hemoglobin, g/dL	0.99	0.985–0.995	<0.001*	-	-	-
Sodium, mmol/L	0.88	0.85–0.90	<0.001*	0.95	0.92–0.98	0.001†
Total cholesterol, mmol/L	0.70	0.63–0.78	<0.001*	0.86	0.77–0.95	0.003†
High density cholesterol, mmol/L	0.36	0.24–0.53	<0.001*	-	-	-
Creatinine, umol/L	1.009	1.007–1.011	<0.001*	-	-	-
Blood urea nitrogen, mg/dL	1.11	1.09–1.12	<0.001*	1.06	1.04–1.08	<0.001†
Uric acid, g/dL	1.002	1.002–1.003	<0.001*	-	-	-
Total bilirubin, umol/L	1.013	1.011–1.015	<0.001*	1.003	0.999–1.006	0.101†
Albumin, g/L	0.90	0.88–0.92	<0.001*	0.97	0.94–0.99	0.005†
C-reactive protein, mg/L	1.007	1.005–1.009	<0.001*	-	-	-
lnNT-proBNP, pg/mL per 1 log unit	2.36	2.10–2.66	<0.001*	1.34	1.15–1.56	<0.001†
lnsST2, ng/mL, per 1 log unit	2.73	2.42–3.07	<0.001	1.52	1.30–1.78	<0.001
sST2 quartiles						
Quartile 2 (vs. quartile 1)	1.67	1.08–2.61	0.023			
Quatiele 3 (vs. quartile 1)	2.54	1.68–3.86	<0.001			
Quartile 4 (vs. quartile 1)	6.92	4.71–10.16	<0.001			

*Variables were included in multivariable analysis (*P*<0.1). †Variables that remained significant in multivariable analysis (*P*<0.05) were added to final multivariable model. The logarithmic functions of NT-proBNP and sST2 were used in the multivariable model.

CI  =  confidence interval; HR  =  hazard ratio; other abbreviation as in Table 1.

Table 3 details results of ROC testing using sST2 to predict events. At baseline the AUC for sST2 did not show significant differences with that of NT-proBNP (all *P* value>0.05). Over time, baseline sST2 concentrations retained ability to classify risk, but sensitivity of sST2 in prediction gradually declined, consistent with evolution of disease in previously lower-risk patients. Lastly, AUC, category-free NRI and IDI analyses indicated significant added predictive value of sST2 to that of NT-proBNP in short and longer term (Table 4).

**Table 3:** The values of sST2 and NT-proBNP for predicting all-cause death and transplantation according to follow-up time.

Follow-up time	AUC (95% CI)*	Cutoff point	Sensitivity	Specificity	PPV	NPV
sST2						
1-month (n = 1528)	0.82 (0.77–0.87)	52.7 ng/mL	0.74	0.75	0.15	0.98
3-month (n = 1528)	0.80 (0.76–0.84)	51.6 ng/mL	0.71	0.76	0.24	0.96
6-month (n = 1392)	0.79 (0.75–0.83)	48.0 ng/mL	0.72	0.74	0.28	0.95
1-year (n = 1244)	0.77 (0.73–0.81)	47.8 ng/mL	0.66	0.75	0.35	0.92
2-year (n = 784)	0.75 (0.71–0.79)	46.7 ng/mL	0.64	0.74	0.39	0.88
3-year (n = 397)	0.76 (0.70–0.81)	48.9 ng/mL	0.62	0.80	0.50	0.87
NT-proBNP						
1-month (n = 1528)	0.79 (0.74–0.83)	1733.3 pg/mL	0.88	0.56	0.10	0.99
3-month (n = 1528)	0.77 (0.73–0.81)	1733.3 pg/mL	0.84	0.58	0.17	0.97
6-month (n = 1392)	0.78 (0.75–0.82)	1733.3 pg/mL	0.84	0.59	0.22	0.96
1-year (n = 1244)	0.77 (0.74–0.81)	1998.9 pg/mL	0.76	0.67	0.32	0.93
2-year (n = 784)	0.75 (0.71–0.79)	1979.0 pg/mL	0.70	0.70	0.38	0.90
3-year (n = 397)	0.73 (0.67–0.79)	1598.2 pg/mL	0.75	0.64	0.40	0.90

AUC  =  area under the curve; CI  =  confidence interval; NPV  =  negative predictive value; NT-proBNP  =  N-terminal pro-B-type natriuretic peptide; PPV  =  positive predictive value.

*All *P* value>0.05 when compared AUC of sST2 with that of NT-proBNP according to follow-up time.

**Table 4:** Improvement of sST2 to NT-proBNP for predicting all-cause death and transplantation according to follow-up time.

Follow-up time	AUC* (95% CI)	ΔAUC (95% CI)	*P* value	category-free NRI (95% CI)	P value	IDI (95% CI)	*P* value
1-month (n = 1528)	0.84 (0.80–0.88)	0.058 (0.024–0.093)	0.001	0.67 (0.40–0.94)	<0.001	0.089 (0.036–0.142)	0.001
3-month (n = 1528)	0.82 (0.78–0.86)	0.052 (0.023–0.080)	<0.001	0.64 (0.43–0.84)	<0.001	0.106 (0.063–0.149)	<0.001
6-month (n = 1392)	0.82 (0.79–0.86)	0.039 (0.017–0.061)	0.001	0.59 (0.40–0.78)	<0.001	0.096 (0.057–0.135)	<0.001
1-year (n = 1244)	0.81 (0.78–0.84)	0.037 (0.017–0.057)	<0.001	0.49 (0.31–0.66)	<0.001	0.069 (0.036–0.103)	<0.001
2-year (n = 784)	0.79 (0.76–0.83)	0.044 (0.019–0.070)	0.001	0.44 (0.25–0.62)	<0.001	0.066 (0.028–0.105)	0.001
3-year (n = 397)	0.79 (0.74–0.84)	0.061 (0.019–0.104)	0.005	0.48 (0.21–0.75)	<0.001	0.080 (0.018–0.142)	0.012

*AUC  =  area under the curve when combined with sST2 and N-terminal pro-B-type natriuretic peptide, all *P* value <0.05 when compared with AUC of sST2;

CI  =  confidence interval; IDI  =  integrated discrimination improvement; NRI  =  net reclassification improvement.

## Discussion

In this cohort of 1528 hospitalized patients with HF, we present the first large scale analysis of sST2 measurement in Asia. These results are especially meaningful because more than half of the study participants were from diffuse geographic regions across China. In contrast to United States (US) registries of hospitalized patients with ADHF [20], [21], patients in our study were much younger (average in our study was 58 years vs. 73 years in US), were mostly male (70.4% in our study vs. 48% in US), and had relatively low systolic blood pressure (average in our study was 119 mmHg vs. 144 mmHg in US). So the results of our study will be important to better understand the characteristics of sST2 on hospitalized patients with HF in China. Therefore, our study was designed to prove and extend the association between sST2 and adverse events.

The process of HF is considered to be accompanied with inflammation. ST2, a member of IL-1 receptor family, had been found to possess immunomodulatory functions, particularly regarding CD4+ T-helper 2 lymphocytes [22]. Beyond this, however, sST2 plays a role in the development of cardiac fibrosis and hypertrophy by modulating IL-32/ST2 signaling system [23]. In response to mechanical stimulation, the transcript for both sST2 and ST2L are up-regulated, and sST2 can act as a decoy receptor for IL-33 competitively inhibiting the cardioprotective function of ST2L [23]. In this context, sST2 appears to be associated with the process of HF progression; thus, sST2 has been found to predict cardiac risk quite potently. With the local availability of a recently developed highly sensitive sST2 assay came the opportunity to specifically examine the role of this biomarker in Asian populations.

We found some notable characteristics of sST2 in our study participants. When categorized by ischemic etiology, sST2 concentrations were higher in patients with non-ischemic HF etiology than patients with ischemic HF etiology, but this may have been due to the fact that the proportion of patients with NYHA functional class IV was higher in non-ischemic groups. Notably, we extended the result of previous study which reported sST2 concentrations were greater in patients with systolic HF than those with HF with preserved ejection fraction (HFpEF) [24], and found sST2 concentrations were not significant different between systolic HF and HFpEF regarding NYHA functional class., In correlation analysis, we found patients with abnormal liver function and low albumin had higher concentrations of sST2; both have been reported to be associated with adverse events of HF [25]–[27]. Interestingly, as a marker of cardiac fibrosis and hypertrophy, sST2 were negatively associated with RWT. This could be explained that sST2 might be associated with excessive ventricular dilatation or involved in the process of apoptosis, both of which resulted in the less thickening of LV wall. The main result of our study was that elevated sST2 levels were associated with adverse events in hospitalized patients with HF. This prognostic value remained robust when adjusted for relevant covariates. Our findings are consistent with the results of previous reports of Western patients with ADHF [7], [8], [10], [24] and chronic HF [11], [14]. After evaluating the association between the prognostic ability of baseline sST2 and time, we found the sensitivity of sST2 in prediction gradually declined. That said, the changes in ability to prognosticate with a single baseline value underscore the importance of serial measurement of sST2, which has been recently shown to add substantial prognostic merit for patients with ADHF and chronic HF. The optimal cut off of sST2 used to discriminate patients with or without death or cardiac transplantation was different from that provided by Ky B, et al. who included 40% patients with NYHA functional class III or IV symptoms [11]. In our study, there was 80.1% of patients was confirmed to be NYHA functional class III or IV on admission. Given the different demography characteristics between these two studies, the optimal cut-off points of sST2 for adverse events might be different. Overtime, we found the prognostic ability of sST2 was similar to that of NT-proBNP, but baseline sST2 concentrations appeared to have more pronounced positive predictive value when compared with those of NT-proBNP, which might indicate the added prognostic value of sST2 to NT-proBNP, supported by discrimination and reclassification analysis.

## Study limitations

Our study has limitations. Firstly, 128 patients were lost during longer term follow up. However, these patients completed the first six months follow-up, and were included in this analysis. Second, we studied patients with both new onset HF as well as decompensation of chronic HF, which might have resulted in mixing cases of different severity and chronicity, with heterogeneity of risk prediction. Third, the model for predicting sST2 concentration showed a low R^2^ of 0.38 meaning that further 62% of effect was unaccounted for. Fourth, no other markers (eg, galectin-3, high sensitivity troponins, growth differentiation factor 15) that have been suggested to be associated with risks of heart failure were available for all patients. We could not assess whether the prognostic value of sST2 beyond these biomarkers in risk prediction. Finally, while our analysis is one of the largest studies of sST2 in HF, the biomarker was not measured serially, which has been suggested to increase prognostic yield [28].

## Conclusions

In summary, sST2 was an independent risk predictor in hospitalized Chinese patients with HF. It also provided additional predictive value to NT-proBNP, and informed substantial reclassification of risk beyond NT-proBNP as well. These first results for sST2 in a uniquely Chinese population will allow for a better understanding of the role played by this important emerging biomarker in a global population.

## Supporting Information

Figure S1 **The flow diagram for patient selection.**
(TIF)

Figure S2 **Geographic distribution of patients across China.**
(TIF)

Table S1 * measured by enzyme-linked immunosorbant assay in a microtiter plate format (Critical Diagnostics, San Diego, US); † measured by the fluorescence immunoassay using the Triage Meter (Alere Inc, San Diego, US); CV  =  coefficient of variation.
(DOC)

Table S2 * Correlations were performed in patients with LVEF ≤40%; NT-proBNP  =  N-terminal pro-B-type natriuretic peptide.
(DOC)

File S1 **Available Data for patients studied.**
(XLSX)
