# Supplementary figures and images for: The Prognostic Value of Plasma Soluble ST2 in Hospitalized Chinese Patients with Heart Failure
Source: PLoS One. 2014 Oct 27;9(10):e110976. doi: 10.1371/journal.pone.0110976 (PMC4210209; doi:10.1371/journal.pone.0110976)

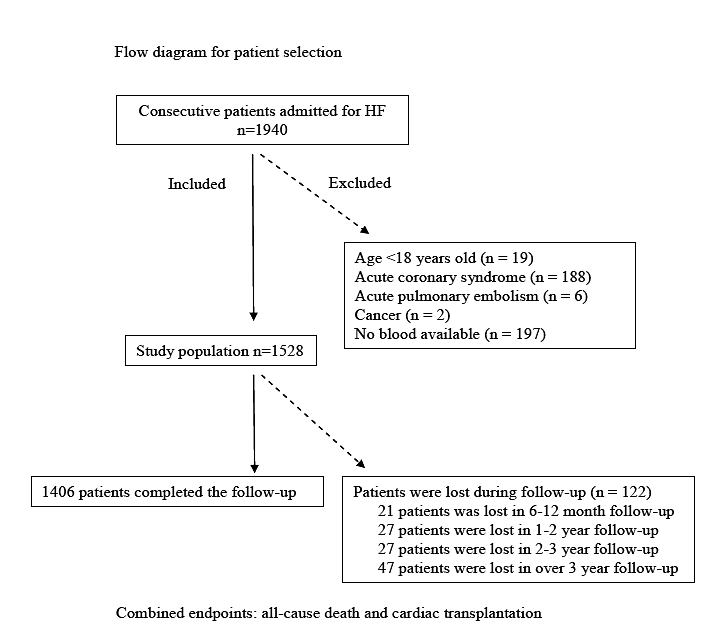

Supplement: Figure S1 — The flow diagram for patient selection. (TIF) [file pone.0110976.s001.tif]

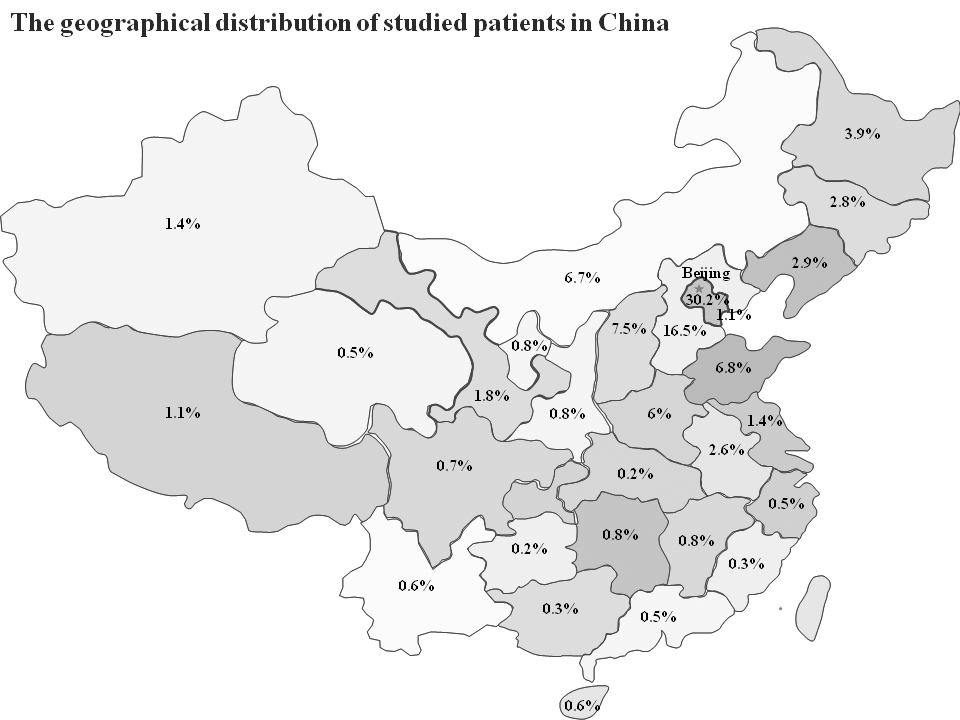

Supplement: Figure S2 — Geographic distribution of patients across China. (TIF) [file pone.0110976.s002.tif]
