# Supplementary material for: The Prognostic Value of Plasma Soluble ST2 in Hospitalized Chinese Patients with Heart Failure
Source: PLoS One. 2014 Oct 27;9(10):e110976. doi: 10.1371/journal.pone.0110976 (PMC4210209; doi:10.1371/journal.pone.0110976)
Supplement: Table S1 — * measured by enzyme-linked immunosorbant assay in a microtiter plate format (Critical Diagnostics, San Diego, US); † measured by the fluorescence immunoassay using the Triage Meter (Alere Inc, San Diego, US); CV = coefficient of variation. (DOC) [file pone.0110976.s003.doc]

**Table S1.**  Characteristics of assay performance

* measured by enzyme-linked immunosorbant assay in a microtiter plate format (Critical Diagnostics, San

|  |  | CV for Intra-assay | |  | CV for Inter-assay | |
| --- | --- | --- | --- | --- | --- | --- |
|  | Range of detection | CV | Number of tests |  | CV | Number of tests |
| sST2* | 3.1-200 ng/ml | 6.4% at 22.5 ng/mL | 25 |  | 4.8% at 22.8 ng/mL | 25 |
|  |  | 5.3% at 36.9 ng/mL | 25 |  | 5.9% at 36.3 ng/mL | 25 |
|  |  | 6.2% at 61.7 ng/mL | 25 |  | 5.7% at 52.5 ng/mL | 25 |
| NT-proBNP† | 20-35000 pg/ml | 9.6% at 1526 pg/ml | 20 |  | 8.4% at 1255 pg/ml | 20 |

Diego, US)

† measured by the fluorescence immunoassay using the Triage® Meter (Alere Inc, San Diego, US)

CV = coefficient of variation
