# Supplementary material for: The Prognostic Value of Plasma Soluble ST2 in Hospitalized Chinese Patients with Heart Failure
Source: PLoS One. 2014 Oct 27;9(10):e110976. doi: 10.1371/journal.pone.0110976 (PMC4210209; doi:10.1371/journal.pone.0110976)
Supplement: Table S2 — * Correlations were performed in patients with LVEF ≤40%; NT-proBNP = N-terminal pro-B-type natriuretic peptide. (DOC) [file pone.0110976.s004.doc]

| **Table S2. Univariable correlations between sST2 and NT-proBNP and selected covariates** | | | | | |
| --- | --- | --- | --- | --- | --- |
|  | sST2 | |  | NT-proBNP | |
| Variable | r | *P* value |  | r | *P* value |
| NT-proBNP, pg/mL | 0.450 | <0.001 |  | - | - |
| Age, yrs | -0.024 | 0.343 |  | 0.053 | 0.038 |
| Systolic blood pressure, mmHg | -0.220 | <0.001 |  | -0.242 | <0.001 |
| Heart rate, beats/min | 0.204 | <0.001 |  | 0.185 | <0.001 |
| Left ventricular ejection fraction,%* | -0.161 | <0.001 |  | -0.343 | <0.001 |
| LV mass index (g/m^2^) | 0.050 | 0.061 |  | 0.264 | <0.001 |
| Relative wall thickness | -0.062 | 0.020 |  | -0.213 | <0.001 |
| Body mass index, kg/m^2^ | -0.140 | <0.001 |  | -0.237 | <0.001 |
| White blood cell count | 0.169 | <0.001 |  | 0.008 | 0.758 |
| Hemoglobin, g/dL | -0.037 | 0.145 |  | -0.130 | <0.001 |
| Albumin, g/L | -0.293 | <0.001 |  | -0.354 | <0.001 |
| Total bilirubin, umol/L | 0.353 | <0.001 |  | 0.301 | <0.001 |
| Sodium, mmol/L | -0.226 | <0.001 |  | -0.112 | <0.001 |
| Creatinine, umol/L | 0.150 | <0.001 |  | 0.245 | <0.001 |
| Blood urea nitrogen, mg/dL | 0.224 | <0.001 |  | 0.300 | <0.001 |
| Uric acid, mg/dL | 0.215 | <0.001 |  | 0.305 | <0.001 |
| Total cholesterol, mmol/L | -0.205 | <0.001 |  | -0.174 | <0.001 |
| High density lipoprotein, mmol/L | -0.175 | <0.001 |  | -0.138 | <0.001 |
| C-reactive protein, mg/L | 0.412 | <0.001 |  | 0.304 | <0.001 |

* Correlations were performed in patients with LVEF ≤ 40%

NT-proBNP = N-terminal pro-B-type natriuretic peptide
